# Maternal obesity shapes associations between preeclampsia and birthweight in pregnancies exposed to low-dose aspirin

**DOI:** 10.3389/fphys.2026.1816077

**Published:** 2026-06-05

**Authors:** Mariano Reynoso, Ana I. Corominas, Franco Solimine, Alberto Ferreiros, Roberto Casale, Alicia E. Damiano

**Affiliations:** 1 Departamento de Ciencias Biológicas, Cátedra de Biología Celular y Molecular, Facultad de Farmacia y Bioquímica, Universidad de Buenos Aires, Buenos Aires, Argentina; 2 Departamento de Fisicomatemática, Cátedra de Matemática, Facultad de Farmacia y Bioquímica, Universidad de Buenos Aires, Buenos Aires, Argentina; 3 Hospital Nacional Prof. A Posadas, El Palomar, Buenos Aires, Argentina; 4 Laboratorio de Biología de la Reproducción, Facultad de Medicina, Instituto de Fisiología y Biofísica Bernardo Houssay (IFIBIO Houssay), Consejo Nacional de Investigaciones Científicas y Técnicas (CONICET)-Universidad de Buenos Aires, Buenos Aires, Argentina

**Keywords:** birthweight, fetal sex, gestational age, low-dose aspirin, maternal obesity, preeclampsia

## Abstract

**Introduction:**

Preeclampsia is a major cause of maternal and perinatal morbidity and is associated with substantial variability in gestational duration and fetal growth. Low-dose aspirin (LDA) is widely used in pregnancies at increased risk, although perinatal outcomes may vary across clinically defined maternal subgroups.

**Methods:**

We conducted a *post hoc* analysis of 284 singleton pregnancies to examine associations between preeclampsia and perinatal outcomes, including gestational age at delivery and neonatal birthweight, and to evaluate whether these associations varied across maternal obesity and LDA exposure strata. Analyses were performed separately in female and male newborns.

**Results:**

Preeclampsia was associated with lower birthweight in both sexes (MD = −747.6 g, p < 0.0001 in females; MD = −867.7 g, p = 0.0127 in males), with a substantial proportion of these differences captured by shorter gestational duration in the mediation model (MD = −2.81 weeks, p < 0.0001 in females; MD = −3.16 weeks, p = 0.0017 in males). Within LDA-exposed pregnancies, no statistically significant differences in birthweight or gestational age relative to the reference profile were observed across obesity strata in male newborns. In female newborns, maternal obesity was associated with lower birthweight (MD = −1002.9 g, p = 0.0003) and shorter gestational age (MD = −2.60 weeks, p = 0.0497), including a non-mediated component estimated within the modeling framework (MD = −506.6 g, p = 0.0043), although estimates in the smallest profiles should be interpreted as exploratory.

**Discussion:**

These findings describe heterogeneity in the associations linking preeclampsia, gestational duration, and fetal growth across maternal obesity and LDA exposure strata. Within this observational framework, the findings support a more contextualized interpretation of perinatal variability across risk-defined clinical profiles.

## 1 Introduction

Preeclampsia remains a leading cause of maternal and perinatal morbidity and mortality worldwide, affecting approximately 5–8% of pregnancies. Despite decades of intensive research, its precise etiology is still not fully understood. Nevertheless, abnormal placentation, exaggerated systemic inflammation, oxidative stress, and widespread endothelial dysfunction are well-recognized hallmarks of the disease (Staff, 2019; Sugulle et al., 2024).

Beyond its role as a major cause of maternal morbidity, preeclampsia has profound consequences for fetal growth and perinatal outcomes (Wilson et al., 2024). Neonatal birthweight represents an integrative marker of intrauterine development, reflecting both gestational duration and the quality of the intrauterine environment. In pregnancies complicated by preeclampsia, reductions in birthweight may arise through multiple, non-mutually exclusive pathways, including placental dysfunction and shortened gestation due to medically indicated early delivery. Emerging evidence further suggests that placental function and fetal adaptive responses to adverse intrauterine environments may differ according to fetal sex, raising the possibility that fetal sex may shape the associations linking preeclampsia, gestational duration, and fetal growth (Clifton, 2010; Santos et al., 2023; Zhang et al., 2024; Smith et al., 2026).

Low-dose aspirin (LDA, acetylsalicylic acid) is currently recommended for the prevention of preeclampsia in women at high risk (Rolnik et al., 2017; Amro et al., 2025). Although its use has been associated with a reduction in disease incidence, the protective effect is modest and far from universal (Mkhize et al., 2021; Walsh and Strauss, 2021; Diguisto et al., 2022; Ninan et al., 2023). This variability likely reflects differences in baseline maternal risk, treatment exposure, and biological responsiveness across clinically defined subgroups. Reported sources include the timing of treatment initiation, aspirin dosage, baseline maternal risk profile, and comorbidities such as obesity, as well as biological factors that may influence aspirin responsiveness. As a result, some women develop preeclampsia despite guideline-based prophylaxis. In this context, maternal obesity and fetal sex have emerged as plausible modifiers of aspirin response, although their combined influence remains insufficiently explored (Kupka et al., 2024; George et al., 2025).

Maternal obesity, an increasingly prevalent global health problem, is characterized by chronic low-grade inflammation, insulin resistance, and metabolic dysregulation. These alterations may exacerbate placental stress and endothelial dysfunction, thereby increasing susceptibility to hypertensive disorders of pregnancy (Zhang et al., 2024). Nevertheless, the interaction between obesity and aspirin efficacy remains controversial. Some studies suggest that maternal body mass index (BMI) has minimal impact on aspirin response, while others report that obesity may reduce aspirin antiplatelet effects, possibly due to aspirin resistance or changes in pharmacodynamics (Cantu et al., 2015; Amro et al., 2025).

While LDA is widely used to prevent preeclampsia, comparatively less attention has been paid to its association with perinatal outcomes in pregnancies that develop the disorder despite prophylaxis. Accordingly, it remains unclear whether aspirin exposure influences the associations between preeclampsia, gestational age at delivery, and neonatal birthweight, or whether these relationships differ according to maternal obesity or fetal sex.

In this context, the aim of the present study was to explore whether maternal obesity and LDA exposure modulate the associations between preeclampsia, gestational age at delivery, and neonatal birthweight in pregnancies exposed to low-dose aspirin. We also examined whether these associations differed according to fetal sex and estimated the relative contribution of gestational age-mediated and non-mediated components to birthweight variation. These findings may help refine the interpretation of aspirin-associated perinatal variability and support more individualized approaches toward personalized obstetric care.

## 2 Materials and methods

### 2.1 Study design and population

This study is a *post hoc* analysis of a single-center cohort conducted at the Hospital Nacional Prof. Dr. Alejandro Posadas between March 2022 and June 2025. The protocol was approved by the institutional ethics committee, and all participants provided written informed consent prior to inclusion.

Pregnant women were screened during the first trimester using the Fetal Medicine Foundation (FMF) risk-prediction algorithm, which integrates clinical, biophysical, and biochemical parameters to estimate the risk of preterm preeclampsia. In accordance with institutional clinical practice, women classified as high risk were prescribed prophylactic low-dose aspirin (LDA, 150 mg/day) between 12 and 16 weeks of gestation, continuing until 36 weeks or delivery, whichever occurred first. Those classified as low risk did not receive aspirin. All clinical management and outcomes occurred independently of the present analysis and without investigator intervention. Accordingly, this study is classified as a non-experimental, observational cohort analysis.

Given the non-randomized allocation of LDA based on FMF risk stratification, LDA exposure reflects clinical risk indication rather than randomized assignment, and comparisons involving LDA-exposed and unexposed groups are subject to confounding by indication.

### 2.2 Inclusion and exclusion criteria

The study included singleton pregnancies with complete clinical and obstetric records, comprising both normotensive and preeclamptic pregnancies.

Preeclampsia was defined according to American College of Obstetricians and Gynecologists (ACOG) criteria (Gestational Hypertension and Preeclampsia: ACOG Practice Bulletin, Number 222, 2020) as new-onset hypertension after 20 weeks of gestation, characterized by systolic blood pressure ≥140 mmHg and/or diastolic blood pressure ≥90 mmHg on at least two occasions, in association with proteinuria (≥300 mg/24 h, protein/creatinine ratio ≥0.3, or dipstick ≥1+) or, in the absence of proteinuria, evidence of end-organ dysfunction, including thrombocytopenia, renal insufficiency, impaired liver function, pulmonary edema, or new-onset cerebral or visual symptoms. Exclusion criteria comprised multiple gestations, chronic kidney disease, liver disease, autoimmune disorders, cardiovascular disease, pregestational or gestational diabetes, other hypertensive disorders of pregnancy, cancer, uterine malformations, assisted reproductive technology pregnancies, oligohydramnios, and polyhydramnios.

### 2.3 Variables and data collection

Clinical and obstetric data were obtained from medical records. Variables included pregnancy group (normotensive or preeclamptic), maternal age at pregnancy onset, pregestational body mass index (BMI), gestational weight gain, and obesity status (defined as BMI ≥ 30 kg/m²). Additionally, data on LDA exposure and timing of initiation, systolic and diastolic blood pressure, proteinuria, gestational age (GA) at delivery, and neonatal birthweight and sex were collected. Data completeness was verified according to predefined inclusion criteria for variables entering the analytical models; no missing values were present for the variables included in the final analysis.

### 2.4 Statistical analysis

In this analytical framework, preeclampsia was treated as the primary exposure variable. Gestational age at delivery and neonatal birthweight were modeled as the primary continuous outcomes. Gestational age was additionally modeled as a mediator of the association between preeclampsia and birthweight within a moderated mediation framework.

#### 2.4.1 Software and general parameters

All statistical analyses were performed using R software (R Core Team) within the RStudio environment (Posit Software, PBC). Graphical outputs were generated with GraphPad Prism (version 8.0.2). Statistical significance was set at α = 0.05.

The full reproducible analysis pipeline, including scripts for data processing, statistical analyses, and model fitting, is publicly available at https://github.com/reynosomariano/lda-pe-reproducible-pipeline. A frozen version corresponding to the manuscript submission is available at https://github.com/reynosomariano/lda-pe-reproducible-pipeline/releases/tag/v1.0.

#### 2.4.2 Baseline clinical and obstetric characteristics of the study population

Baseline characteristics were summarized across four groups defined by pregnancy group (normotensive vs preeclamptic) and low-dose aspirin (LDA) exposure. Continuous variables were expressed as median [interquartile range], and categorical variables as absolute and relative frequencies.

Global comparisons across the four groups were performed using the Kruskal–Wallis test for continuous variables and Fisher’s exact test for categorical variables. Gestational age at initiation of LDA exposure was compared only between treated groups.

Pairwise comparisons were conducted only for gestational age at delivery and birthweight, using the Conover–Iman procedure with Holm correction for multiple testing, to support descriptive characterization of group differences.

Birthweight was analyzed separately for female and male newborns and compared across groups within each sex.

All statistical tests were two-sided. This analysis was conducted as an initial descriptive step prior to subsequent modeling procedures.

#### 2.4.3 Maternal profile construction

Pregnancy group, LDA exposure, and maternal obesity were treated as dichotomous variables throughout the study, using the following categorical labels: normotensive (N) vs. preeclamptic (PE); unexposed (LDA–) vs. exposed (LDA+); and non-obese (OB–: BMI < 30 kg/m²) vs. obese (OB+: BMI ≥ 30 kg/m²).

By combining these three factors, eight mutually exclusive maternal profiles were constructed to represent distinct clinical contexts. The profile defined by normotensive non-obese pregnancies without LDA exposure (N/LDA–/OB–) served as the reference category for all comparisons.

These profiles and categorical labels were applied consistently across all figures, tables, and statistical models. All primary analyses were stratified by fetal sex to characterize within-sex patterns of association. Formal between-sex comparisons were not included in the primary analytical framework and were evaluated only in complementary sensitivity analyses using multi-group models.

Since LDA was prescribed based on first-trimester risk stratification rather than random assignment, comparisons between profiles are subject to confounding by indication and should be interpreted as associations within clinically defined contexts rather than as evidence of causal effects.

#### 2.4.4 Preterm delivery and low birthweight percentiles

Two perinatal outcomes were evaluated separately for female and male newborns: (i) delivery before 37 gestational weeks and (ii) birthweight below the 10^th^ percentile for sex- and gestational age-specific references. For each outcome, maternal profiles were compared against the reference profile (N/LDA–/OB–). Relative risk (RR), attributable risk (AR), and their 95% percentile confidence intervals were estimated using non-parametric bootstrap (10, 000 resamples). Differences in proportions were assessed using Fisher’s exact test, and p-values were adjusted using the Benjamini–Hochberg method to control the false discovery rate (FDR) in exploratory multiple comparisons.

In contingency tables with zero-cell counts, the Haldane–Anscombe correction (addition of 0.5 to all cells) was applied to allow stable estimation of relative risks and corresponding confidence intervals, avoiding undefined or infinite estimates.

#### 2.4.5 Birthweight distribution and global comparisons across maternal profiles

Mean birthweight and associated variability measures (standard deviation, standard error, and 95% percentile confidence intervals) were estimated for each maternal profile using non-parametric bootstrap resampling (10, 000 iterations), separately for female and male newborns.

Global differences in birthweight across the eight maternal profiles were assessed using a permutation-based one-way ANOVA (10, 000 resamples). When the global test was significant, *post hoc* contrasts were conducted within the same permutation framework, focusing on mean differences (MD) between each maternal profile and the reference profile (N/LDA−/OB−). These MD were estimated along with their corresponding p-values, which were adjusted using the Benjamini–Hochberg procedure to control the false discovery rate (FDR) in exploratory multiple comparisons.

#### 2.4.6 Moderated mediation via structural equation modeling

To characterize how pregnancy group, maternal obesity, and LDA exposure are jointly associated with birthweight through gestational age, a moderated mediation framework was implemented using Structural Equation Modeling (SEM). Models were fitted separately for female and male newborns using the lavaan package in R, with the normotensive, non-obese, LDA-unexposed profile (N/LDA–/OB–) serving as the reference category for all contrasts (MacKinnon, 2008).

The model included two linked equations specifying gestational age as mediator and birthweight as outcome. Pregnancy group (preeclampsia vs normotensive), LDA exposure, and maternal obesity were included as main effects and through their pairwise and three-way interactions in both equations.

Gestational age at delivery was modeled as:


$$GA_{i}=\alpha_{0}+a_{1}PE_{i}+a_{2}LDA_{i}+a_{3}OB_{i}+a_{12}(PE_{i}\times LDA_{i})+a_{13}(PE_{i}\times OB_{i})+a_{23}(LDA_{i}\times OB_{i})+a_{123}(PE_{i}\times LDA_{i}\times OB_{i})+\epsilon_{GA_{i}}$$

Birthweight was modeled as:


$$BW_{i}=\beta_{0}+c_{1}PE_{i}+c_{2}LDA_{i}+c_{3}OB_{i}+c_{12}(PE_{i}\times LDA_{i})+c_{13}(PE_{i}\times OB_{i})+c_{23}(LDA_{i}\times OB_{i})+c_{123}(PE_{i}\times LDA_{i}\times OB_{i})+bGA_{i}+\epsilon_{BW_{i}}$$

A detailed description of model parameterization and notation is provided in the **Supplementary Methods** (see **Supplementary Methods S1**).

Indirect effects were estimated as the product of the profile-specific effect on gestational age and the gestational age–birthweight association (path *b*), allowing decomposition into gestational-age–mediated and non-mediated components. A common gestational age–birthweight association (path *b*) was assumed within each fetal-sex stratum. This assumption was evaluated through interaction testing between gestational age and pregnancy group, LDA exposure, and maternal obesity, as well as through sensitivity analyses assessing alternative model specifications (see **Supplementary Methods S3.2**).

For each fetal sex, associations were decomposed into total, gestational-age–mediated (indirect), and non–gestational-age–mediated (direct) components. Models were estimated using maximum likelihood, and uncertainty was quantified using non-parametric bootstrap resampling (10, 000 iterations), with percentile-based 95% confidence intervals.

**Figure 1:**
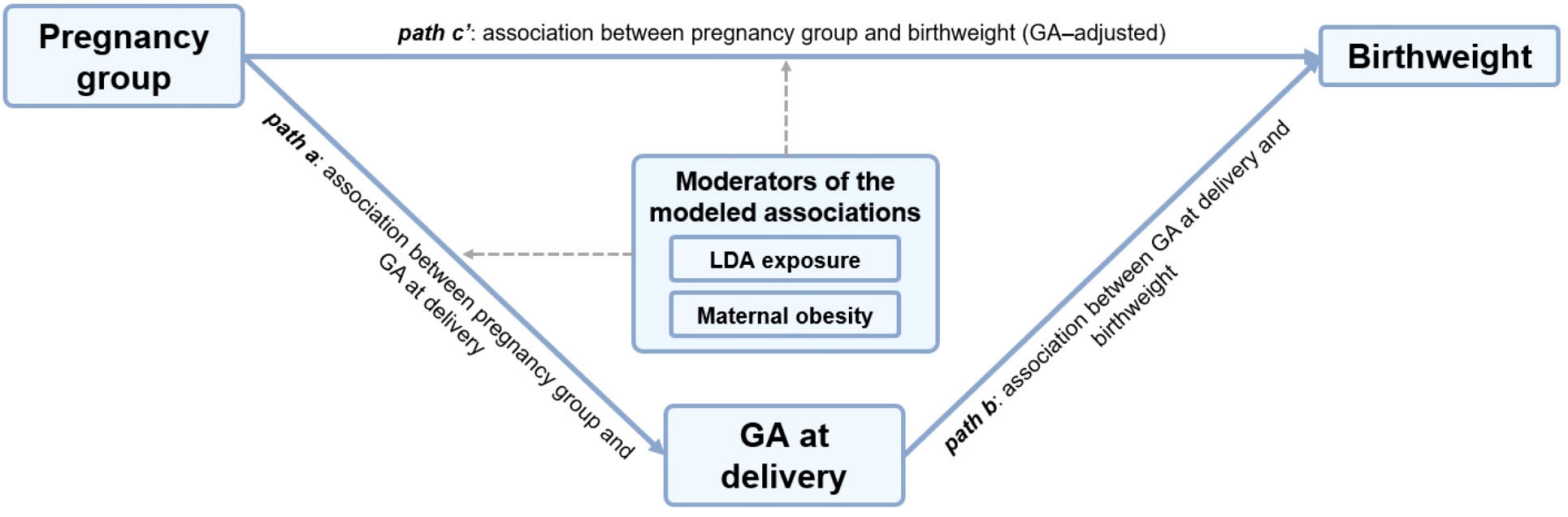
Conceptual diagram of the moderated mediation framework used to decompose associations between pregnancy group and neonatal birthweight into gestational age–mediated and non-mediated components. Pregnancy group (normotensive vs. preeclamptic) was specified as the focal predictor, gestational age (GA) at delivery as the mediator, and birthweight as the outcome. The pathway linking pregnancy group to birthweight (path c′), the pathway linking pregnancy group to gestational age (path a), and the pathway linking gestational age to birthweight (path b) are depicted. Low-dose aspirin (LDA) exposure and maternal obesity were included as moderators of the modeled associations between the pregnancy group and both gestational age and birthweight. Dashed arrows indicate moderated pathways. Models were fitted separately for female and male newborns.

Results are expressed as profile-anchored contrasts, with each maternal profile compared against the reference category (N/LDA–/OB–). The conceptual structure of the moderated mediation model is shown in **Figure 1**.

#### 2.4.7 Analytical hierarchy and multiplicity

Analyses were conducted using a predefined hierarchical strategy to prioritize interpretability and reduce overfitting. The analytical sequence began with descriptive and stratified comparisons, followed by multivariable modeling and structural analyses to evaluate associations among preeclampsia, gestational age at delivery, and neonatal birthweight. Additional methodological details regarding inferential hierarchy and multiplicity are provided in the **Supplementary Methods S2**.

#### 2.4.8 Sensitivity analyses

Sensitivity analyses were performed to evaluate the robustness of model-derived estimates in maternal profiles with limited sample size and to assess the stability of key modeling assumptions. For small-sample maternal profiles (n ≤ 6), robustness of SEM-derived components was examined using leave-one-out and bootstrap-based directional stability analyses. In addition, sensitivity analyses were conducted to evaluate the assumption of a common gestational age–birthweight association (path *b*) across maternal profiles through comparison of progressively less constrained SEM specifications. Full implementation details are provided in the **Supplementary Methods S3**.

#### 2.4.9 Multi-group structural modeling

As a complementary analysis, multi-group SEM was performed using fetal sex as the grouping variable to evaluate potential heterogeneity in structural parameters across sexes. These analyses were conducted as a complementary assessment of parameter heterogeneity and were not part of the primary inferential framework. Full model specifications and comparison procedures are provided in the **Supplementary Methods S4**.

## 3 Results

### 3.1 Study population

A total of 284 singleton pregnancies were included, comprising 237 (83.5%) normotensive and 47 (16.5%) preeclamptic cases. Among normotensive pregnancies, 82 (34.6%) had received low-dose aspirin (LDA), whereas 21 (44.7%) of preeclamptic pregnancies had been exposed to LDA. In this cohort, LDA exposure reflected first-trimester FMF risk stratification, with LDA-exposed pregnancies corresponding to those classified as high risk. Within the preeclamptic group, 10 cases (21.3%) corresponded to early-onset disease (<34 weeks), of which 7 (70.0%) occurred in pregnancies not exposed to LDA.

Among LDA-exposed pregnancies, the median gestational week at treatment initiation was 13 weeks in normotensive pregnancies and 14 weeks in preeclamptic pregnancies, with no significant global differences (p = 0.0922).

Median maternal age ranged from 28 to 31 years across the four clinical groups (N/LDA–, N/LDA+, PE/LDA–, and PE/LDA+), with no significant global differences (p = 0.6196).

The proportion of maternal obesity differed globally across the four groups (p = 0.0010), with higher values observed in LDA-exposed pregnancies, particularly among those with preeclampsia. Pregestational BMI also differed globally (p = 0.0114), with the highest values observed in preeclamptic pregnancies exposed to LDA. Gestational weight gain ranged from a median of 7.0 to 13.0 kg across groups and did not differ significantly (p = 0.7132).

Systolic and diastolic blood pressure differed significantly across groups (p < 0.0001), with higher values observed in preeclamptic pregnancies. Proteinuria was detected exclusively in preeclamptic pregnancies.

Gestational age at delivery differed globally across the four groups (p < 0.0001). Pairwise comparisons indicated lower gestational age in preeclamptic pregnancies without LDA (36.3 weeks, p < 0.0001) and with LDA (37.9 weeks, p < 0.0001), compared with normotensive pregnancies without LDA.

The distribution of fetal sex did not differ across groups (p = 0.4004).

When birthweight was analyzed separately by fetal sex, global differences were detected within each sex across the four groups. In female newborns, the global difference was significant (p < 0.0001), with lower birthweight observed in preeclamptic pregnancies both with LDA exposure (2834.5 g, p < 0.0001) and without LDA exposure (2420.0 g, p < 0.0001), compared with normotensive pregnancies without LDA. In male newborns, global differences were also observed (p = 0.0067). Lower birthweight was detected only in preeclamptic pregnancies without LDA exposure (2950.0 g, p = 0.0117) relative to normotensive pregnancies without LDA (**Table 1**).

**Table 1:** Maternal and perinatal characteristics of normotensive and preeclamptic pregnancies according low-dose aspirin exposure.

Variables		Normotensive pregnancies		Preeclamptic pregnancies		p-value (overall)
		LDA–	LDA+	LDA–	LDA+	
n		155	82	26	21	
Maternal age (years)		28 [24 to 33]	29 [25 to 34]	28 [23 to 33]	31 [25 to 34]	0.6196
Pregestational BMI (kg/m^2^)		27.2 [23.8 to 29.5]	27.6 [23.5 to 33.8]	27.0 [23.5 to 30.1]	33.1 [26.3 to 37.3]	0.0114
Maternal obesity (%)		23.2	45.1	26.9	52.4	0.0010
Maternal weight gain (kg)		12.0 [8.2 to 15.7]	7.0 [4.5 to 12.6]	13.0 [7.2 to 19.0]	9.5 [6.9 to 13.8]	0.7132
Initiation of LDA exposure (weeks)		—	13.0 [12.0 to 14.0]	—	14.0 [13.0 to 16.0]	0.0922
Systolic blood pressure (mmHg)		120.0 [117.5 to 120.0]	120.0 [120.0 to 120.0]	160 [140.0 to 170.0]	150.0 [140.5 to 160.0]	<0.0001
Diastolic blood pressure (mmHg)		70.0 [70.0 to 80.0]	70.0 [70.0 to 75.0]	100.0 [90.0 to 100.0]	92.5 [90.0 to 98.8]	<0.0001
Proteinuria^#^		Negative	Negative	Positive	Positive	—
Gestational age at delivery (weeks)		39.0 [38.1 to 39.4]	38.4 [38.0 to 39.0]	36.3 [34.2 to 37.9]*	37.9 [37.0 to 38.7]*	<0.0001
Fetal sex (%)	Female	49.7	47.6	65.4	57.1	0.4004
	Male	50.3	52.4	34.6	42.9	
Birthweight (g)	Female	3385.0 [3072.0 to 3713.0]	3050.0 [2779.5 to 3375.0]*	2420.0 [2103.0 to 3184.0]*	2834.5 [1949.5 to 2972.2]*	<0.0001
	Male	3377.5 [3197.2 to 3730.0]	3305.0 [2813.5 to 3570.5]	2950.0 [1258.0 to 3095.0]*	3145.0 [2800.0 to 3444.0]	0.0067

Continuous variables, including maternal age, pregestational BMI, maternal weight gain, gestational age at initiation of LDA exposure, systolic blood pressure, diastolic blood pressure, gestational age at delivery, and birthweight are presented as median and interquartile range [IQR]. Global comparisons across the groups were performed using the Kruskal–Wallis test. Gestational age at initiation of LDA exposure was compared only between LDA-exposed pregnancies. Birthweight is shown separately for female and male newborns and was compared across the groups within each sex. Pairwise comparisons were performed only for gestational age at delivery and birthweight using the Conover–Iman procedure with Holm correction for multiple testing when global differences were detected. Significant pairwise differences are indicated with an asterisk (*) and refer to comparisons versus normotensive pregnancies without LDA exposure (LDA–).

Categorical variables, including maternal obesity and fetal sex are expressed as percentages and compared across the groups using Fisher’s exact test.

“—” indicates not applicable.

^#^ Proteins in urine were assessed using the Test Urine Labstix Strip.

Baseline maternal characteristics across the eight maternal profiles defined by pregnancy group, LDA exposure, and maternal obesity are provided in **Supplementary Table 1** to facilitate assessment of potential baseline differences across groups.

### 3.2 Preterm delivery in female and male newborns

In normotensive pregnancies without LDA exposure, maternal obesity was not associated with a higher frequency of preterm delivery (<37 weeks) in either female or male newborns (**Tables 2A, B**). By contrast, among normotensive non-obese pregnancies with LDA exposure, preterm delivery was observed in female (13.6%, p = 0.0293) and male (17.4%, p = 0.0124) newborns. In the presence of maternal obesity, this pattern was attenuated in females but remained evident in males (15.0%, p = 0.0262) (**Table 2B**).

**Table 2:** Risk estimates for delivery before 37 gestational weeks across maternal profiles.

Profile	n	<37 (%)	≥37 (%)	RR [95% CI]	AR [95% CI] (pp)	p(raw)	p(BH)
A. Female newborns							
N/LDA–/OB+	16	0.0	100.0	3.7 [3.7 to 3.7]	0.0 [0.0 to 0.0]	1.0000	1.0000
N/LDA+/OB–	22	13.6	86.4	18.8 [2.7 to 35.0]	13.6 [0.0 to 27.3]	0.0168	0.0293
N/LDA+/OB+	17	5.9	94.1	10.3 [3.4 to 24.1]	5.9 [0.0 to 17.6]	0.2179	0.2543
PE/LDA–/OB–	14	64.3	35.7	78.5 [45.5 to 103.3]	64.3 [35.7 to 85.7]	<0.0001	<0.0001
PE/LDA–/OB+	3	33.3	66.7	46.5 [15.5 to 108.5]	33.3 [0.0 to 100.0]	0.0469	0.0656
PE/LDA+/OB–	6	33.3	66.7	44.3 [8.7 to 79.7]	33.3 [0.0 to 66.7]	0.0068	0.0158
PE/LDA+/OB+	6	33.3	66.7	44.3 [8.7 to 79.7]	33.3 [0.0 to 66.7]	0.0068	0.0158
B. Male newborns							
N/LDA–/OB+	20	0.0	100.0	2.8 [2.8 to 2.8]	0.0 [0.0 to 0.0]	1.0000	1.0000
N/LDA+/OB–	23	17.4	82.6	22.1 [7.4 to 41.8]	17.4 [4.4 to 34.8]	0.0053	0.0124
N/LDA+/OB+	20	15.0	85.0	19.7 [2.8 to 36.5]	15.0 [0.0 to 30.0]	0.0150	0.0262
PE/LDA–/OB–	5	60.0	40.0	68.8 [29.5 to 108.7]	60.0 [20.0 to 100.0]	0.0003	0.0018
PE/LDA–/OB+	4	50.0	50.0	59.0 [11.8 to 106.2]	50.0 [0.0 to 100.0]	0.0032	0.0111
PE/LDA+/OB–	4	25.0	75.0	35.4 [11.8 to 82.6]	25.0 [0.0 to 75.0]	0.0645	0.0903
PE/LDA+/OB+	5	0.0	100.0	9.8 [9.8 to 9.8]	0.0 [0.0 to 0.0]	1.0000	1.0000

Relative risk (RR), attributable risk (AR, pp: percentage points), and their 95% bootstrap confidence intervals (CI, 10, 000 resamples) for the probability of delivery before 37 gestational weeks across maternal profiles. Profiles were constructed based on pregnancy group, LDA-exposure and maternal obesity status. Analyses were conducted separately for female (A, n = 145) and male (B, n = 139) newborns, with all profiles compared against a common reference group N/LDA–/OB–.

Differences in proportions were evaluated using Fisher’s exact test, with raw and Benjamini–Hochberg-adjusted (BH) p-values reported.

When no events were observed in any of the profiles being compared, RR estimates remained computable due to the Haldane–Anscombe correction; in these situations, the bootstrap CI collapses to identical bounds matching the corrected RR value. For AR, the point estimate becomes 0, and its bootstrap CI accordingly collapses to 0.0 to 0.0. These collapsed intervals reflect absence of information rather than absence of association.

In preeclamptic pregnancies, preterm delivery was markedly more frequent in both sexes. In the absence of LDA exposure, preterm birth occurred at similarly high frequencies in female (64.3%, p < 0.0001) and male (60.0%, p = 0.0018) pregnancies.

Among LDA-exposed preeclamptic pregnancies, residual preterm delivery remained evident in female newborns (**Table 2A**), including both non-obese (33.3%, p = 0.0158) and obese maternal profiles (33.3%, p = 0.0158). In male newborns, preterm delivery was less consistently observed across the corresponding maternal profiles, including no preterm births in obese pregnancies (**Table 2B**).

### 3.3 Low birthweight in female and male newborns

In normotensive pregnancies, the frequency of low birthweight (<10^th^ percentile) remained low and did not differ across groups defined by LDA exposure or maternal obesity in either female or male newborns (**Tables 3A, B**).

**Table 3:** Risk estimates for birthweight below the 10^th^ percentile across maternal profiles.

Profile	n	<P10 (%)	≥P10 (%)	RR [95% CI]	AR [95% CI] (pp)	p(raw)	p(BH)
A. Female newborns							
N/LDA–/OB+	16	0.0	100.0	3.7 [3.7 to 3.7]	0.0 [0.0 to 0.0]	1.0000	1.0000
N/LDA+/OB–	22	9.1	90.9	13.5 [2.7 to 29.7]	9.1 [0.0 to 22.7]	0.0679	0.1568
N/LDA+/OB+	17	0.0	100.0	3.4 [3.4 to 3.4]	0.0 [0.0 to 0.0]	1.0000	1.0000
PE/LDA–/OB–	14	14.3	85.7	20.7 [4.1 to 45.5]	14.3 [0.0 to 35.7]	0.0328	0.1567
PE/LDA–/OB+	3	33.3	66.7	46.5 [15.5 to 108.5]	33.3 [0.0 to 100.0]	0.0469	0.1567
PE/LDA+/OB–	6	0.0	100.0	8.9 [8.9 to 8.9]	0.0 [0.0 to 0.0]	1.0000	1.0000
PE/LDA+/OB+	6	16.7	83.3	26.6 [8.9 to 62.0]	16.7 [0 to 50.0]	0.0896	0.1568
B. Male newborns							
N/LDA–/OB+	20	0.0	100.0	2.8 [2.8 to 2.8]	0.0 [0.0 to 0.0]	1.0000	1.0000
N/LDA+/OB–	23	8.7	91.3	12.3 [2.5 to 27.0]	8.7 [0.0 to 21.7]	0.0781	0.1367
N/LDA+/OB+	20	5.0	95.0	8.4 [2.8 to 19.7]	5.0 [0.0 to 15.0]	0.2564	0.3584
PE/LDA–/OB–	5	40.0	60.0	49.2 [9.8 to 88.5]	40.0 [0.0 to 80.0]	0.0051	0.0179
PE/LDA–/OB+	4	25.0	75.0	35.4 [11.8 to 82.6]	25.0 [0.0 to 75.0]	0.0645	0.1367
PE/LDA+/OB–	4	0.0	100.0	11.8 [11.8 to 11.8]	0.0 [0.0 to 0.0]	1.0000	1.0000
PE/LDA+/OB+	5	40.0	60.0	49.2 [9.8 to 88.5]	40.0 [0.0 to 80.0]	0.0051	0.0179

Relative risk (RR), attributable risk (AR, pp: percentage points), and their 95% bootstrap confidence intervals (CI, 10, 000 resamples) for the probability of having a birthweight below the 10^th^ percentile for sex and gestational age across maternal profiles. Profiles were constructed based on pregnancy group, LDA-exposure and maternal obesity status. Analyses were conducted separately for female (A, n = 145)3 and male (B, n = 139) newborns, with all profiles compared against a common reference group (N/LDA–/OB–).

Differences in proportions were evaluated using Fisher’s exact test, with raw and Benjamini–Hochberg-adjusted (BH) *p*-values reported.

When no events were observed in any of the profiles being compared, RR estimates remained computable due to the Haldane–Anscombe correction; in these situations, the bootstrap CI collapses to identical bounds matching the corrected RR value. For AR, the point estimate becomes 0, and its bootstrap CI accordingly collapses to 0.0 to 0.0. These collapsed intervals reflect absence of information rather than absence of association.

By contrast, in preeclamptic pregnancies, the distribution of low birthweight showed distinct patterns across fetal sex. In male newborns, preeclampsia was associated with a higher frequency of birthweight below the 10^th^ percentile (40.0%, p = 0.0179). Lower frequencies were observed in LDA-exposed pregnancies, whereas higher frequencies were observed in the presence of maternal obesity, reaching values comparable to those observed in preeclamptic pregnancies without LDA exposure (40.0%, p = 0.0179) (**Table 3B**). In female newborns, the proportion classified below the 10^th^ percentile did not differ significantly across maternal profiles, despite consistent reductions in mean birthweight (**Table 3A**).

### 3.4 Birthweight as a continuous outcome in female and male newborns

**Figure 2:**
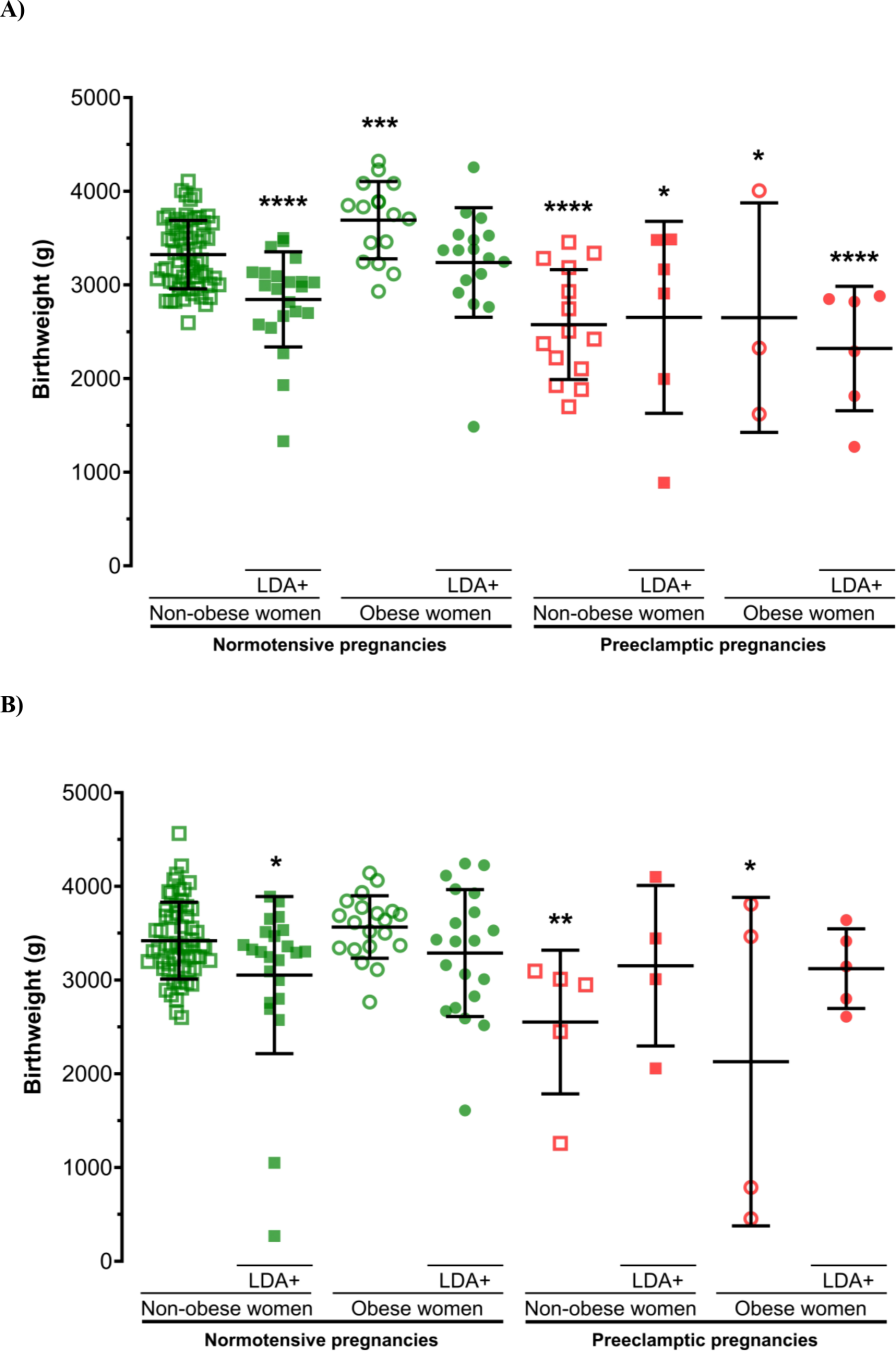
Birthweight distribution across maternal profiles defined by pregnancy group, LDA exposure, and maternal obesity status, stratified by fetal sex. **(A)** Female newborns, **(B)** Male newborns. Each dot represents one individual birthweight observation (grams). Green symbols correspond to normotensive pregnancies and red symbols to preeclamptic pregnancies; empty symbols indicate absence of LDA exposure, and filled symbols indicate its presence; squares represent newborns from non-obese women and circles from obese women. Horizontal lines represent mean ± SD. Overall differences across maternal profiles were evaluated separately within each sex using a one-way permutation ANOVA (10, 000 permutations). *Post hoc* comparisons were restricted to contrasts against the reference profile (N/LDA–/OB–) within each sex, using permutation-based tests. P-values were adjusted for multiple comparisons using the Benjamini–Hochberg false discovery rate procedure. Significance levels are indicated as adjusted p < 0.05 (*), p < 0.01 (**), p < 0.001 (***), and p < 0.0001 (****).

Analysis of birthweight as a continuous variable revealed different patterns of association across maternal characteristics within independently analyzed fetal-sex strata (**Figure 2**). In normotensive pregnancies, maternal obesity was associated with a higher mean birthweight in female newborns compared with the reference profile (MD = 367.7 g, p = 0.0044), whereas no relevant differences were observed in males (MD = 146.0 g, p = 0.1551). Among normotensive non-obese pregnancies, mean birthweight differed across LDA exposure categories in both female (MD = −478.5 g, p < 0.0001) and male (MD = −367.0 g, p = 0.0482) newborns, whereas in pregnancies of obese women exposed to LDA, mean birthweight did not differ from the reference profile in either sex.

By contrast, preeclampsia was associated with a pronounced reduction in birthweight in both female (MD = −747.6 g, p < 0.0001) and male (MD = −867.7 g, p = 0.0056) newborns. In female newborns, this reduction remained evident in pregnancies with maternal obesity (MD = −673.3 g, p = 0.0034). In male newborns, lower mean birthweight was mainly observed in preeclamptic pregnancies of non-obese women, whereas in pregnancies with maternal obesity, estimates were more imprecise and differences relative to normotensive pregnancies within the same stratum were not consistent.

Across LDA exposure strata, additional differences were observed within independently analyzed fetal-sex groups. In male newborns, no relevant differences in mean birthweight were observed in LDA-exposed preeclamptic pregnancies, either in non-obese women (MD = −267.3 g, p = 0.4006) or obese women (MD = −298.3 g, p = 0.2815). In female newborns, LDA-exposed preeclamptic pregnancies were associated with lower mean birthweight both in the absence of maternal obesity (MD = −669.9 g, p = 0.0120) and, more markedly, in obese women (MD = −1002.9 g, p < 0.0001).

### 3.5 Moderated mediation analysis

**Figure 3:**
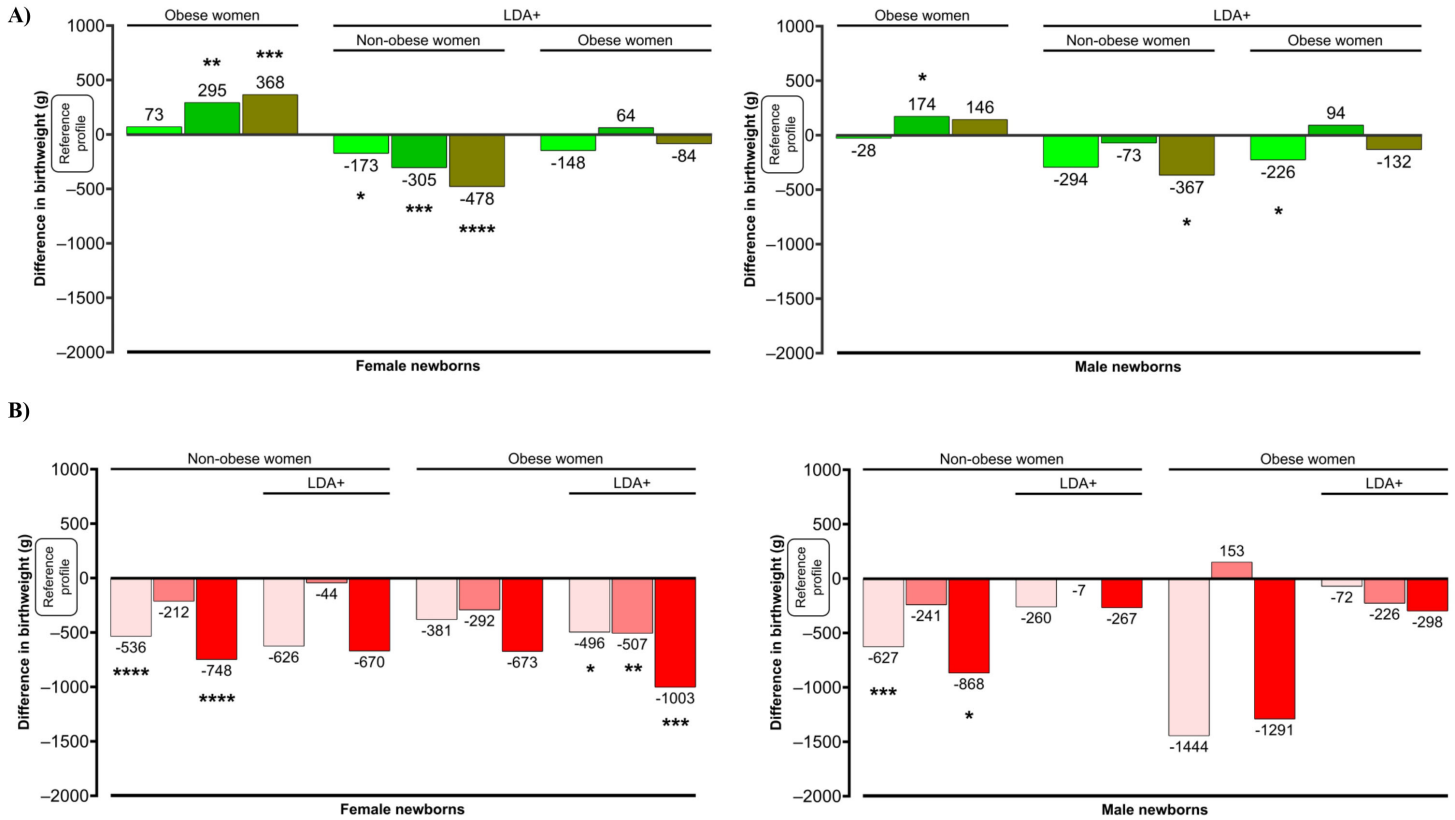
Decomposition of birthweight differences across maternal profiles into gestational age–mediated and non-mediated components, stratified by fetal sex. **(A)** Normotensive pregnancies, **(B)** Pregnancies complicated by preeclampsia. Bars represent the estimated differences in birthweight (grams) for each maternal profile relative to the reference profile (N/LDA–/OB–). Within each maternal profile, bars are displayed from left to right with increasing color intensity, representing the gestational age–mediated component, the gestational age–independent component, and the total modeled association. Components in **(A)** are shown using a green color scale (light green: GA–mediated component; green: GA–independent component; dark green: total association), whereas components in **(B)** are shown using a red color scale (light pink: GA–mediated component; light red: GA–independent component; red: total association). Estimates were obtained using a moderated mediation framework implemented via Structural Equation Modeling, with statistical inference based on non-parametric bootstrap resampling (10, 000 resamples). Statistical significance of profile-specific contrasts relative to the reference category was evaluated using bootstrap-based p-values and is indicated as p < 0.05 (*), p < 0.01 (**), p < 0.001 (***), and p < 0.0001 (****).

Within this framework, the association between preeclampsia and birthweight was decomposed into a component not mediated by gestational age and a gestational age–mediated component. In normotensive pregnancies, decomposition of profile-anchored associations relative to the reference profile (N/LDA–/OB–) revealed distinct patterns according to maternal obesity and LDA exposure (**Figure 3A**). The higher mean birthweight observed in normotensive pregnancies with maternal obesity in female newborns was mainly explained by the non-mediated component (MD = 294.5 g, p = 0.0055), with no evidence of a relevant gestational age–mediated contribution. In male newborns, the non-mediated component showed the largest contribution (MD = 173.7 g, p = 0.0366), whereas the gestational age–mediated component was small and not statistically significant, and the overall association did not differ significantly from the reference profile.

In normotensive pregnancies with LDA exposure, the pattern of associations differed descriptively across sex-stratified analyses. In non-obese normotensive women, lower mean birthweight was observed in both female and male newborns. In female newborns, this reduction reflected contributions from both the gestational age–mediated component (MD = −173.3 g, p = 0.0427) and the non-mediated component (MD = −305.2 g, p = 0.0004), suggesting that part of the association was accounted for by variation in gestational age (36.2%, p = 0.0112), relative to the reference profile (N/LDA–/OB–) within the model framework.

In male newborns, a lower mean birthweight was also observed (MD = −366.9 g, p = 0.0415), although neither the gestational age–mediated nor the non-mediated component reached statistical significance when considered separately.

In obese normotensive pregnancies with LDA exposure, no significant overall associations with birthweight were detected relative to the reference profile, despite the presence of a gestational age–mediated component in male newborns (MD = −226.5 g, p = 0.0203).

In preeclamptic pregnancies, substantial negative associations with birthweight were observed in both sexes (**Figure 3B**). In female newborns, the total mean difference relative to the reference profile was −747.6 g (95% CI: −1065.5 to −421.1; p < 0.0001). A substantial proportion of this association was accounted for within the model by shorter gestational age at delivery. Preeclampsia was associated with a mean shift of −2.81 gestational weeks (p < 0.0001), corresponding to a gestational age–mediated component of −536.0 g (p < 0.0001), accounting for 71.7% of the total birthweight difference (p < 0.0001).

In male newborns, the total mean difference relative to the reference profile was −867.7 g (95% CI: −1623.0 to −341.5; p = 0.0127). A substantial proportion of this association was also captured through the gestational age–mediated component. The gestational age shift was −3.16 weeks (p = 0.0017), yielding a mediated component of −626.9 g (p = 0.0039), accounting for 72.3% of the total birthweight difference (p = 0.0021).

Associations involving LDA exposure varied according to maternal obesity status in sex-stratified analyses.

In non-obese preeclamptic pregnancies with LDA exposure, neither the total association with birthweight nor its gestational age–mediated or non-mediated components reached statistical significance relative to the reference profile in either sex. In contrast, in obese preeclamptic pregnancies, negative associations with birthweight were observed in sex-stratified analyses, with different patterns across strata.

In male newborns of obese preeclamptic women with LDA exposure, no significant differences were observed in gestational age at delivery or in either the mediated or non-mediated components of birthweight relative to the reference profile.

In female newborns of obese preeclamptic women with LDA exposure, however, a reduction in gestational age was observed (−2.6 weeks, p = 0.0497), yielding a gestational age–mediated component of −496.3 g (95% CI: −1056.5 to −135.4, p = 0.0474), accounting for 49.5% of the total birthweight difference (p = 0.0019), with the remaining contribution captured by a significant non-mediated component (MD = −506.6 g, 95% CI: −886.3 to −202.0, p = 0.0043).

These findings describe variation in the modeled associations linking preeclampsia, maternal obesity, gestational age, and birthweight across sex-stratified LDA-exposed clinical profiles.

Formal multi-group SEM comparisons showed no statistically significant evidence of sex-related heterogeneity in structural parameters between female and male newborns (**Supplementary Table 3**). Accordingly, differences observed across sex-stratified analyses should be interpreted as descriptive variation in modeled associations rather than as evidence of statistically significant effect modification by fetal sex.

Sample size across maternal profiles within each fetal-sex stratum is shown in **Supplementary Table 2**.

Sensitivity analyses restricted to small-sample profiles showed high directional consistency of model-derived components across leave-one-out and bootstrap-based perturbation analyses, with reduced stability confined to the smallest profiles (**Supplementary Tables 4**, **S5**).

Evaluation of the model specification indicated no evidence of heterogeneity in the gestational age–birthweight association across maternal profiles in female newborns (**Supplementary Table 6**). In male newborns, the permutation-based block test suggested potential interaction effects between gestational age and maternal profiles (**Supplementary Table 6**). However, sensitivity analyses using progressively relaxed SEM specifications showed that allowing subgroup-specific variation in path b resulted in unstable estimates and reduced precision, despite some improvement in local fit indices (**Supplementary Table 8**). Bootstrap-based profile-specific slope estimation showed wide and overlapping confidence intervals across maternal profiles, supporting the lack of robust evidence for stable subgroup-specific slope differences (**Supplementary Table 7**). Importantly, the direction of the gestational age–birthweight association remained consistent across all specifications.

## 4 Discussion

The present study indicates that associations linking preeclampsia, gestational duration, and birthweight vary across maternal profiles defined by low-dose aspirin (LDA) exposure and maternal obesity. By integrating sex-stratified analyses with a moderated mediation framework, we observed different patterns in gestational length and birthweight across LDA-exposed and unexposed profiles within each sex, highlighting variation in these associations according to maternal metabolic status.

Our results also contribute to current understanding of how LDA exposure relates to preterm delivery and low birthweight below clinical cutoffs (<37 weeks, <10^th^ percentile). While lower frequencies of preterm birth were observed in certain LDA-exposed subgroups, patterns for low birthweight were less uniform, with variation observed across maternal obesity strata within each sex. These observations are broadly consistent with findings from meta-analyses reporting that early initiated LDA is associated with reduced rates of preterm preeclampsia and related neonatal adverse outcomes (Wang and Chang, 2025).

Importantly, normotensive pregnancies exposed to LDA in this cohort should not be interpreted as low-risk or physiologically normal gestations. LDA was prescribed based on first-trimester risk stratification, indicating an underlying predisposition to preeclampsia that did not ultimately manifest clinically. Therefore, all analyses involving LDA exposure should be interpreted within a profile-anchored associational framework, in which exposure reflects underlying clinical risk rather than experimentally assigned treatment, and estimated effects represent conditional associations rather than causal treatment effects. Nonetheless, these pregnancies remain clinically informative because they represent individuals identified as high risk in early gestation who did not subsequently develop overt preeclampsia, thereby providing a relevant comparator for interpreting profile-specific associations under risk-based LDA exposure.

Within the SEM framework, a substantial proportion of the association between preeclampsia and birthweight was accounted for by the indirect component through gestational age. Different patterns were observed in sex-stratified analyses. In male newborns, birthweight differences were largely accounted for by earlier delivery. In female newborns, a non–gestational age–mediated component was also observed, suggesting additional growth-related mechanisms beyond gestational shortening. This was consistent with a dissociation between continuous and categorical growth assessments. Reductions in mean birthweight among females were not consistently captured by low-birthweight classification, whereas in male newborns birthweight more frequently crossed clinical thresholds. These findings underscore the limitations of relying solely on categorical cutoffs to characterize fetal growth alterations.

Several subgroup-specific estimates were derived from profiles with ≤6 observations and should therefore be interpreted as exploratory, despite generally stable directional behavior in sensitivity analyses.

The patterns observed in sex-stratified analyses may be interpreted in the context of emerging evidence that placental function and fetal adaptation to adverse intrauterine environments may differ according to fetal sex. Male fetuses may prioritize growth and nutrient transfer with limited adaptive flexibility under hypoxic or inflammatory stress, potentially increasing vulnerability to earlier delivery. In contrast, female fetuses may adopt more adaptive metabolic and inflammatory responses that favor survival but are associated with restricted growth (Clifton, 2010; Eriksson et al., 2010; Dalton-O’Reilly et al., 2025).

Although the gestational age–birthweight association was constrained to be equal across maternal profiles within each sex, formal testing and sensitivity analyses supported this specification in female newborns. In male newborns, some evidence of heterogeneity was observed; however, relaxing this constraint resulted in unstable and poorly interpretable estimates, supporting the use of a common path b as a parsimonious model structure. This modeling choice implies that indirect (gestational age–mediated) effects are interpreted under the assumption of a shared gestational age–birthweight relationship within each sex, which is important for the interpretation of the mediation decomposition.

Maternal obesity was identified as a clinically and biologically relevant factor in the observed associations, rather than being treated solely as a covariate. Obese women with preeclampsia exhibited more pronounced reductions in gestational length and birthweight. In sex-stratified analyses, these reductions were more apparent in female newborns, consistent with the patterns described above. Obesity has been consistently linked to impaired placental perfusion, heightened oxidative stress, endothelial dysfunction, and dysregulated prostanoid pathways, all of which may interact with aspirin-sensitive mechanisms (Saben et al., 2014; Kelly et al., 2020; Santos et al., 2023; Louwen et al., 2024; Zhang et al., 2024). In addition, expanded plasma volume, increased platelet turnover, and incomplete COX-1 acetylation in obesity have been proposed to reduce LDA bioavailability and pharmacodynamic responsiveness, potentially attenuating aspirin-associated benefit in risk-defined clinical settings (Finneran et al., 2019). Within this altered metabolic and inflammatory environment, placental adaptations that differ according to fetal sex may further shape aspirin-associated patterns. Male placentas may be more vulnerable to disruptions in mitochondrial and vascular function associated with earlier delivery, whereas female placentas may preferentially activate stress-response and inflammatory programs that modify pathways relevant to aspirin-responsive vascular and thrombotic regulation (Bucher et al., 2021; Santos et al., 2023; Dalton-O’Reilly et al., 2025).Taken together, these mechanisms provide a coherent biological framework to interpret the patterns observed in this study, highlighting the potential relevance of maternal metabolic status and fetal sex as contextual factors and supporting the need for further investigation in study designs that can formally evaluate their role in risk stratification and preventive strategies.

These biologically plausible patterns further reinforce the relevance of disentangling mediated and non-mediated pathways linking preeclampsia, maternal phenotype, and fetal growth. Beyond its analytical role, the moderated mediation framework enabled the separation of gestational age–mediated and non–gestational age–mediated components within the association between preeclampsia and birthweight reduction.

This distinction suggests that part of the observed birthweight reduction is closely linked to gestational duration and may therefore be more relevant to clinical contexts in which pregnancy prolongation is achievable. Conversely, the component not explained by gestational age may be interpreted as reflecting underlying placental dysfunction, which remains less amenable to current preventive strategies.

This analytical distinction provides additional conceptual clarity when interpreting fetal growth alterations in preeclampsia, separating effects driven primarily by delivery timing from those reflecting underlying placental pathology.

From a translational perspective, our findings suggest that perinatal associations observed under standard 150 mg/day LDA exposure vary according to maternal metabolic status, with patterns observed in sex-stratified analyses. These observations should not be interpreted as evidence of altered aspirin pharmacologic efficacy in obese pregnancies, but rather as clinically heterogeneous associations observed under risk-based aspirin exposure. Although emerging pharmacodynamic evidence has raised the possibility of altered aspirin responsiveness in obesity, clinical trials evaluating weight-adjusted dosing remain limited and inconclusive (Cantu et al., 2015; Poon et al., 2017; Finneran et al., 2019; Amro et al., 2025). Our observations are compatible with a precision medicine framework for preeclampsia prevention in which maternal BMI, placental biomarkers, and fetal sex could be evaluated as candidate factors for risk stratification models. Within this context, future studies should determine whether stratified preventive approaches, including variation in dose or timing, improve clinical utility in specific high-risk subgroups identified through evolving early screening algorithms.

This study benefits from a well-characterized clinical cohort and, to our knowledge, is one of the few to apply a moderated mediation framework to examine the influence of maternal obesity and LDA exposure on pregnancy outcomes within sex-stratified analyses. This approach enabled the examination of gestational age–mediated and non-mediated components linking maternal phenotype, LDA exposure, and fetal outcomes. Nonetheless, its *post hoc* design, the absence of objective measures of aspirin adherence or biochemical response, and the moderate overall sample size may limit statistical power.

In addition, because LDA prescription was based on clinical risk stratification rather than random allocation, residual confounding by indication cannot be excluded and may have contributed to the observed profile-specific associations. In particular, stratification across multiple biological modifiers (preeclampsia status, LDA exposure, maternal obesity, and fetal sex) resulted in smaller subgroup cells, which may affect the stability and precision of certain interaction and mediation estimates.

Although sensitivity analyses supported the general directional robustness of the main modeled components, estimates derived from the smallest profiles were less stable and should be interpreted as exploratory.

An additional limitation relates to the specification of a common gestational age–birthweight association (path b) across maternal profiles. Although some variability in this relationship cannot be excluded, subgroup-specific estimation proved unstable in sensitivity analyses and did not consistently improve model fit. The use of a common path b was therefore retained as a parsimonious modeling strategy to preserve stability and interpretability. This implies that mediation estimates rely on a shared gestational age–birthweight relationship within each sex, which should be considered when interpreting indirect effects.

Multi-group SEM analyses did not provide statistically significant evidence of sex-related heterogeneity in model parameters, although descriptive differences were observed across sex-stratified profiles. Accordingly, differences observed across sex-stratified analyses should be interpreted as descriptive patterns rather than as evidence of statistically significant interaction by fetal sex. However, the absence of statistically significant parameter heterogeneity in the multi-group framework does not preclude biologically meaningful sex-related patterning at the descriptive level, particularly in the context of limited subgroup size and reduced power to detect higher-order interaction effects. While formal between-sex parameter heterogeneity was not supported, these descriptive differences remain biologically interpretable and hypothesis-generating in light of known sexually dimorphic placental adaptive responses.

Additionally, the model assumes a linear and homogeneous association between gestational age and birthweight within each sex. This simplifying specification was adopted to ensure model tractability and interpretability. Given that fetal growth is inherently non-linear, this approximation may not fully capture more complex developmental trajectories, and results should be interpreted accordingly.

In summary, the present findings identify heterogeneous associations linking preeclampsia, gestational duration, and fetal growth across maternal profiles defined by LDA exposure and metabolic status. Although formal sex-related heterogeneity was not detected, descriptive variation across sex-stratified clinical profiles supports further investigation in larger cohorts designed for multidimensional risk stratification. These findings should be interpreted as biologically plausible, profile-anchored associations within an observational framework and as hypothesis-generating rather than evidence of differential aspirin efficacy across maternal subgroups. Nonetheless, they support continued investigation into stratified preventive approaches and more context-aware precision medicine frameworks for preeclampsia risk assessment and prevention.

## Data Availability

The data analyzed in this study is subject to the following licenses/restrictions: The datasets generated and analyzed during the current study are not publicly available due to patient confidentiality and institutional regulations but are available from the corresponding author upon reasonable request. Requests to access these datasets should be directed to AD, adamiano@ffyb.uba.ar.
