# Supplementary material for: Maternal obesity shapes associations between preeclampsia and birthweight in pregnancies exposed to low-dose aspirin
Source: Front Physiol. 2026 Jun 5;17:1816077. doi: 10.3389/fphys.2026.1816077 (PMC13278928; doi:10.3389/fphys.2026.1816077)
Supplement: Supplementary file 1 [file DataSheet1.pdf]

SUPPLEMENTARY METHODS

S1. Expanded structural modeling details

To further characterize how pregnancy group, maternal obesity, and low-dose aspirin (LDA) exposure were jointly associated with birthweight through gestational age, structural models were specified to decompose birthweight variation into gestational age–related and non-gestational components. Gestational age was treated as an intermediate variable within the structural framework, reflecting its role in differentiating variation attributable to timing of delivery from variation more directly related to fetal growth.

Maternal obesity and LDA exposure were incorporated as moderators through interaction terms with pregnancy group in both the mediator and outcome equations, allowing the association between preeclampsia and perinatal outcomes to vary across combinations of obesity status and LDA exposure. A mean structure was included to enable estimation of conditional contrasts relative to the reference profile.

The coefficient relating gestational age to birthweight (path *b*) was constrained to be equal across moderator-defined subgroups within each fetal-sex stratum. This specification was based on empirical evaluation under the primary model assumptions and was further examined through sensitivity analyses using alternative model specifications (see Supplementary Methods S3).

Indirect components were estimated as the product of the profile-specific association with gestational age and the common gestational age–birthweight association. The proportion of the total association attributable to gestational age was estimated for each maternal profile. Models were estimated using maximum likelihood, and bootstrap inference was used to improve robustness of interval estimation under moderate sample size and potential departures from normality.

In this framework, the indirect (gestational age–mediated) component quantifies the portion of the association between preeclampsia and birthweight that operates through gestational age at delivery, whereas the direct (non–gestational age–mediated) component represents the remaining association not accounted for by gestational age.

Gestational age at delivery (GA) was modeled as:

1. $GA_i = \alpha_0 + a_1 PE_i + a_2 LDA_i + a_3 OB_i + a_{12}(PE_i \times LDA_i) + a_{13}(PE_i \times OB_i) + a_{23}(LDA_i \times OB_i) + a_{123}(PE_i \times LDA_i \times OB_i) + \varepsilon_{GA_i}$

Birthweight (BW) was modeled as:

2. $BW_i = \beta_0 + c_1 PE_i + c_2 LDA_i + c_3 OB_i + c_{12}(PE_i \times LDA_i) + c_{13}(PE_i \times OB_i) + c_{23}(LDA_i \times OB_i) + c_{123}(PE_i \times LDA_i \times OB_i) + bGA_i + \varepsilon_{BW_i}$

Where:

i: indexes individual pregnancies

GA_i: gestational age at delivery for pregnancy i
BW_i: birthweight for pregnancy i
PE_i: pregnancy group indicator for pregnancy i
0 = normotensive pregnancy (N), 1 = preeclampsia (PE)
LDA_i: low-dose aspirin exposure indicator for pregnancy i
0 = unexposed (LDA –), 1 = exposed (LDA +)
OB_i: maternal obesity indicator for pregnancy i
0 = non-obese (OB –), 1 = obese (OB +)

α_0: intercept for the mediator equation (baseline gestational age)
β_0: intercept for the outcome equation (baseline birthweight)

a_1, a_2, a_3: main effects of PE_i, LDA_i, and OB_i on gestational age, respectively.
c_1, c_2, c_3: main effects of PE_i, LDA_i, and OB_i on birthweight, respectively.

a_{12}, a_{13}, a_{23}: two-way interaction effects corresponding to $PE_i \times LDA_i$, $PE_i \times OB_i$, and $LDA_i \times OB_i$, respectively, on gestational age.
c_{12}, c_{13}, c_{23}: two-way interaction effects corresponding to $PE_i \times LDA_i$, $PE_i \times OB_i$, and $LDA_i \times OB_i$, respectively, on birthweight.
a_{123}: three-way interaction ($PE_i \times LDA_i \times OB_i$) effects on gestational age.
c_{123}: three-way interaction effects on birthweight.

b: effect of gestational age on birthweight (path b), representing the expected change in birthweight associated with a one-unit increase in gestational age, conditional on the other predictors in the model.

ε_{GA_i}: residual error term for gestational age.
ε_{BW_i}: residual error term for birthweight.

All coefficients represent adjusted associations, conditional on the other variables included in the model.

The inclusion of GA_i in the outcome equation ensures that birthweight differences are estimated conditional on gestational age, enabling decomposition into gestational-age–mediated and non-mediated components.

S2. Analytical hierarchy and multiplicity

Analyses followed a predefined inferential hierarchy. Comparative analyses of preterm delivery and birthweight distributions were considered exploratory and supportive, whereas primary inferential emphasis was placed on the SEM path-analytic framework. Model parameters were interpreted as model-based associational components conditional on maternal profile definition, rather than as counterfactual causal effects. Interpretation of results followed this hierarchy, with the SEM framework providing the principal basis for inference.

For exploratory analyses involving multiple pairwise comparisons, including comparisons of proportions across maternal profiles and post hoc contrasts following permutation-based ANOVA, p-values were adjusted using the Benjamini–Hochberg false discovery rate (FDR) procedure. In contrast, the SEM-based moderated mediation analysis was specified *a priori* and estimated within a single jointly fitted model. Accordingly, SEM-derived parameters were interpreted as components of a unified model-based inferential structure, and no additional multiple testing correction was applied.

S3. Expanded sensitivity analyses

S3.1 Extended sensitivity analyses for small-sample maternal profiles

To evaluate the robustness of SEM-derived estimates in maternal profiles with limited sample size ($n \leq 6$), two complementary sensitivity procedures were applied within each fetal-sex stratum.

First, a leave-one-out (LOO) analysis was performed in which each observation within small-sample profiles was sequentially removed and the SEM re-estimated. For each indirect and total component, the range of re-estimated values and the proportion of iterations preserving the original direction (sign) were recorded. This proportion was used as an index of directional stability under single-observation perturbation.

Second, bootstrap-based sign stability analysis was performed using 2,000 non-parametric resamples. For each bootstrap iteration, the SEM was re-estimated and the indirect and total components recalculated. For each component, the proportion of bootstrap estimates preserving the sign of the original point estimate was calculated as an index of directional robustness under resampling-based perturbation.

These complementary procedures were used to evaluate the sensitivity of model-derived estimates to individual observations and resampling variability independently of effect magnitude.

S3.2 Sensitivity analysis of the common gestational age–birthweight association (path *b*)

To evaluate the assumption that the gestational age–birthweight association (path *b*) was constant across maternal profiles, additional sensitivity analyses were performed within each fetal-sex stratum.

First, interaction terms between gestational age and pregnancy group, LDA exposure, and maternal obesity were tested in the birthweight equation using a permutation-based block test (Freedman–Lane approach) to assess whether profile-specific variation in path *b* improved model fit relative to the primary common-slope specification.

Second, profile-specific gestational age–birthweight slopes were estimated using non-parametric bootstrap resampling (2,000 iterations) within each maternal profile and fetal-sex stratum to assess between-profile variability in slope magnitude and direction.

Third, a hierarchical series of progressively less constrained SEM specifications was fitted within each fetal-sex stratum. These alternative models sequentially relaxed the equality constraint on path *b*, allowing subgroup-specific variation according to pregnancy group, LDA exposure, and maternal obesity. Models were estimated using maximum likelihood with non-parametric bootstrap resampling (10,000 iterations) to derive percentile-based confidence intervals. Model fit, parameter stability, and interpretability of the mediated decomposition were compared across specifications.

Together, these analyses were used to assess whether relaxing the common path *b* assumption materially altered model fit, parameter stability, or interpretation of the decomposition framework.

S4. Multi-group structural modeling details

As a complementary analysis, multi-group structural equation modeling (SEM) was performed using fetal sex as the grouping variable to formally assess potential heterogeneity in model parameters across sexes.

A series of nested models was specified to evaluate parameter invariance between female and male newborns. In the fully unconstrained model, all structural paths were freely estimated within each sex. Equality constraints were then imposed sequentially across specific parameter blocks, including: (i) the gestational age–birthweight association (path *b*), (ii) the mediator model coefficients (*a* paths), (iii) the outcome model coefficients (*c* paths), and (iv) a fully constrained model in which all structural parameters were held equal across sexes.

Model comparisons were conducted using robust likelihood ratio tests based on the MLR estimator, complemented by Akaike (AIC) and Bayesian (BIC) information criteria. A significant deterioration in model fit following equality constraints was interpreted as evidence of sex-related heterogeneity in the corresponding parameter block.

Parameter estimates from the unconstrained model were additionally examined descriptively to characterize sex-related differences in effect magnitude and configuration. These analyses were conducted as a complementary assessment of parameter heterogeneity and were not part of the primary inferential framework.
