# Supplementary material for: Maternal obesity shapes associations between preeclampsia and birthweight in pregnancies exposed to low-dose aspirin
Source: Front Physiol. 2026 Jun 5;17:1816077. doi: 10.3389/fphys.2026.1816077 (PMC13278928; doi:10.3389/fphys.2026.1816077)
Supplement: Supplementary file 2 [file DataSheet2.pdf]

SUPPLEMENTARY MATERIAL

Supplementary Table S1. Baseline maternal characteristics across the eight maternal profiles defined by pregnancy group, low-dose aspirin exposure, and maternal obesity

Profile	n	Maternal age (years)	Pregestational BMI (kg/m^2)	Gestational weight gain (kg)	Gestational age at LDA initiation (weeks)
N / LDA– / OB–	119	28 [24-32]	25.6 [23.0-27.2]	12.0 [8.6-17.0]	—
N / LDA– / OB+	36	30 [25-34]	33.3 [30.8-34.8]	12.0 [6.0-13.5]	—
N / LDA+ / OB–	45	27 [23-34]	23.6 [21.2-25.0]	10.0 [8.1-13.0]	13 [12-14]
N / LDA+ / OB+	37	30 [27-34]	34.9 [31.8-39.5]	7.0 [4.5-12.6]	13 [12-14]
PE / LDA– / OB–	19	28 [23-35]	24.4 [22.4-27.2]	18.5 [12.3-22.0]	—
PE / LDA– / OB+	7	28 [24-31]	34.2 [32.5-35.4]	9.5 [7.2-12.8]	—
PE / LDA+ / OB–	10	32 [30-33]	26.2 [23.9-27.1]	12.8 [8.1-17.4]	13 [13-16]
PE / LDA+ / OB+	11	26 [24-33]	36.9 [35.3-43.3]	9.5 [8.8-10.2]	14 [12-16]

Maternal profiles were defined by pregnancy group (normotensive [N] vs preeclampsia [PE]), low-dose aspirin exposure (LDA− vs LDA+), and maternal obesity status (OB− vs OB+). Continuous variables are presented as median [interquartile range]. Gestational age at LDA initiation is shown only for LDA-exposed profiles; "—" indicates not applicable. This table is provided to facilitate the assessment of baseline differences across profiles in this observational study.

Supplementary Table S2. Sample size across maternal profiles by fetal sex

Fetal sex	Profile	n	Small-sample profile
Female	PE / LDA− / OB+	3	Yes
Female	PE / LDA+ / OB−	6	Yes
Female	PE / LDA+ / OB+	6	Yes
Female	PE / LDA− / OB−	14	No
Female	N / LDA− / OB+	16	No
Female	N / LDA+ / OB+	17	No
Female	N / LDA+ / OB−	22	No
Female	N / LDA− / OB−	61	No
Male	PE / LDA− / OB+	4	Yes
Male	PE / LDA+ / OB−	4	Yes
Male	PE / LDA− / OB−	5	Yes

Male	PE / LDA+ / OB+	5	Yes
Male	N / LDA− / OB+	20	No
Male	N / LDA+ / OB+	20	No
Male	N / LDA+ / OB−	23	No
Male	N / LDA− / OB−	58	No

Maternal profiles were defined by pregnancy group (normotensive [N] vs preeclampsia [PE]), low-dose aspirin exposure (LDA− vs LDA+), and maternal obesity status (OB− vs OB+). Profiles with $n \leq 6$ were classified as small-sample profiles and were specifically examined in sensitivity analyses.

Supplementary Table S3. Formal evaluation of sex-related heterogeneity using multi-group structural equation modeling (SEM)

Parameter constraint comparison	$\Delta\chi^2$	Δdf	p-value	AIC (restricted)	BIC (restricted)	AIC (free)	BIC (free)
Path b equal vs free	0.12	1	0.7255	5457.53	5592.54	5459.40	5598.06
Paths a equal vs free	9.98	7	0.1899	5462.44	5575.56	5459.40	5598.06
Paths c equal vs free	7.07	7	0.4215	5452.90	5566.02	5459.40	5598.06
Global model equal vs free	18.27	15	0.2490	5454.07	5538.00	5459.40	5598.06

Multi-group SEM analyses were conducted to formally assess potential heterogeneity in structural parameters across fetal sex. Nested models were compared by constraining parameter blocks (paths a, b, c, and the full model) to be equal across groups. No statistically significant differences were observed for any parameter block (all $p > 0.1899$), indicating no evidence of sex-related heterogeneity in the estimated structural relationships.

Supplementary Table S4. Leave-one-out robustness analysis within small-sample profiles

Fetal sex	Component (indirect or total)	Profile	Full estimate	Min (LOO)	Max (LOO)	% same sign (LOO)
Female	Indirect	PE / LDA− / OB+	-380.97	-713.11	86.06	66.67
Female	Indirect	PE / LDA+ / OB−	-626.20	-742.76	-262.23	100.00
Female	Indirect	PE / LDA+ / OB+	-496.32	-599.01	-259.61	100.00
Female	Total	PE / LDA− / OB+	-673.26	-1350.76	-158.26	100.00
Female	Total	PE / LDA+ / OB−	-669.93	-836.26	-316.66	100.00
Female	Total	PE / LDA+ / OB+	-1002.93	-1114.86	-792.86	100.00
Male	Indirect	PE / LDA− / OB−	-626.92	-712.47	-432.59	100.00
Male	Indirect	PE / LDA− / OB+	-1444.26	-1900.48	-927.36	100.00
Male	Indirect	PE / LDA+ / OB−	-260.23	-377.14	-152.17	100.00
Male	Indirect	PE / LDA+ / OB+	-72.20	-116.01	-16.45	100.00

Male	Total	PE / LDA− / OB−	-867.69	-1003.29	-544.04	100.00
Male	Total	PE / LDA− / OB+	-1290.79	-1850.96	-732.63	100.00
Male	Total	PE / LDA+ / OB−	-267.29	-582.96	97.71	75.00
Male	Total	PE / LDA+ / OB+	-298.29	-427.79	-170.29	100.00

Leave-one-out (LOO) analysis was performed within small-sample profiles (n ≤ 6). Each observation was sequentially removed and the model re-estimated. "Full estimate" represents the component value obtained from the complete dataset. "Min" and "Max" indicate the range of estimates across LOO re-estimations. "% same sign" represents the proportion of iterations in which the direction (sign) of the component remained unchanged, providing a measure of robustness to individual observations.

Supplementary Table S5. Bootstrap-based directional stability analysis within small-sample profiles

Fetal sex	Component (indirect or total)	Profile	Full estimate	% positive	% negative	% same sign
Female	Indirect	PE / LDA− / OB+	-380.97	23.89	76.11	76.11
Female	Total	PE / LDA− / OB+	-673.26	15.20	84.80	84.80
Female	Indirect	PE / LDA+ / OB−	-626.20	0.10	99.90	99.90
Female	Total	PE / LDA+ / OB−	-669.93	4.32	95.68	95.68
Female	Indirect	PE / LDA+ / OB+	-496.32	0.31	99.69	99.69
Female	Total	PE / LDA+ / OB+	-1002.93	0.00	100.00	100.00
Male	Indirect	PE / LDA− / OB+	-1444.26	2.08	97.92	97.92
Male	Total	PE / LDA− / OB+	-1290.79	9.92	90.08	90.08
Male	Indirect	PE / LDA+ / OB−	-260.23	4.67	95.33	95.33
Male	Total	PE / LDA+ / OB−	-267.29	26.95	73.05	73.05
Male	Indirect	PE / LDA− / OB−	-626.92	0.00	100.00	100.00
Male	Total	PE / LDA− / OB−	-867.69	0.00	100.00	100.00
Male	Indirect	PE / LDA+ / OB+	-72.20	15.68	84.32	84.32
Male	Total	PE / LDA+ / OB+	-298.29	6.75	93.25	93.25

Sign stability was assessed using bootstrap resampling (2,000 iterations). "Full estimate" represents the component value obtained from the complete dataset. The proportions of bootstrap samples yielding positive or negative estimates are reported. "% same sign" indicates the proportion of bootstrap samples in which the direction (sign) of the component remained consistent with the original estimate, providing a measure of robustness of directional conclusions.

Supplementary Table S6. Permutation-based block test for heterogeneity in the gestational age-birthweight association

Fetal sex	B	RSS (reduced model)	RSS (full model)	ΔRSS	Relative improvement	p-value
Female	10,000	19,211,630	18,445,222	766,408	0.0399	0.5669
Male	10,000	20,559,839	18,151,418	2,408,421	0.1170	0.0182

Reduced model: birthweight equation with a common gestational age slope. Full model: birthweight equation allowing interactions between gestational age and pregnancy group, LDA exposure, and maternal obesity. ΔRSS = RSS (reduced model) – RSS (full model). Relative improvement = ΔRSS / RSS (reduced model). P-values were obtained using a permutation-based block test (Freedman-Lane approach).

Supplementary Table S7. Bootstrap estimates of gestational age-birthweight slopes across clinical profiles

Fetal sex	Profile	**Slope** (g/week)	95% CI lower	95% CI upper	Includes zero
Female	N / LDA– / OB–	96.0	-28.0	205.0	Yes
Female	N / LDA– / OB+	159.0	-3.9	336.0	Yes
Female	N / LDA+ / OB–	187.0	87.7	302.0	No
Female	N / LDA+ / OB+	242.0	-7.04	467.0	Yes
Female	PE / LDA– / OB–	170.0	-29.5	315.0	Yes
Female	PE / LDA− / OB+	49.4	-243.0	619.0	Yes
Female	PE / LDA+ / OB−	247.0	-5.8	571.0	Yes
Female	PE / LDA+ / OB+	343.0	-248.0	1032.0	Yes
Male	N / LDA– / OB–	142.0	37.0	263.0	No
Male	N / LDA– / OB+	187.0	-76.8	391.0	Yes
Male	N / LDA+ / OB–	181.0	70.6	253.0	No
Male	N / LDA+ / OB+	267.0	161.0	484.0	No
Male	PE / LDA– / OB–	265.0	-389.0	1369.0	Yes
Male	PE / LDA− / OB+	124.0	-1073.0	242.0	Yes
Male	PE / LDA+ / OB−	467.0	-764.0	952.0	Yes
Male	PE / LDA+ / OB+	258.0	-438.0	1040.0	Yes

Slopes represent bootstrap-based estimates of the gestational age-birthweight association within each maternal profile. Confidence intervals are wide and frequently include zero, indicating substantial uncertainty in subgroup-specific estimates. The absence of a consistent pattern of effect modification and the high variability of slopes suggest that subgroup-specific estimation is not reliable, supporting the use of a common gestational age-birthweight association.

Supplementary Table S8. Sensitivity analysis in male newborns: model fit across SEM specifications with progressively relaxed path b

Model	Specification of path b	Converged	χ^2	df	p-value	CFI	TLI	RMSEA	AIC	BIC

M0	Common slope	Yes	0.00	0	—	1.000	1.000	0.000	2717.3	2773.1
M1	EG × EMB	Yes	44.62	2	<0.001	0.979	0.648	0.560	2716.4	2775.1
M2	EG × EMB + TX + OB	Yes	329.76	3	<0.001	0.912	0.387	0.880	2719.3	2783.9
M3	Fully moderated	Yes	366.38	7	<0.001	0.951	0.797	0.608	2714.0	2790.3

Models were estimated in male newborns only. M0 assumes a common gestational age-birthweight association; M1-M3 progressively relax this constraint. Although some indices (e.g., CFI) remained acceptable in partially relaxed models, others (notably RMSEA and TLI) indicated poor fit. No consistent improvement in global fit was observed as model complexity increased.
